# P-1743. Impact of a carbapenem centered antimicrobial stewardship program in the antibiotic consumption patterns and healthcare infections in a tertiary public hospital in Colombia

**DOI:** 10.1093/ofid/ofae631.1906

**Published:** 2025-01-29

**Authors:** Gerardo A Muñeton Lopez, Maria Juliana Amaya, David Suarez, Hernan Vergara

**Affiliations:** Subred Integrada de Servicios de Salud Sur Occidente E.S.E, Bogotá, Distrito Capital de Bogota, Colombia; Hospital MIlitar Central de Bogotá, Bogota, Distrito Capital de Bogota, Colombia; Hospital MIlitar Central de Bogotá, Bogota, Distrito Capital de Bogota, Colombia; Universidad Militar Nueva Granada, Bogota, Distrito Capital de Bogota, Colombia

## Abstract

**Background:**

Antimicrobial stewardship programs (ASP) recommend the control of multiple antibiotics related to bacterial resistance in order to prevent the induction of ESBL, carbapenemases, and vancomycin resistance. Nevertheless, the control of all these antibiotics sometimes is not possible due to the limited workforce in the ASP team. In this study, we decided to limit all ASP interventions to the control of carbapenems, leaving the intervention of other antibiotics as a secondary task

**Figure:**
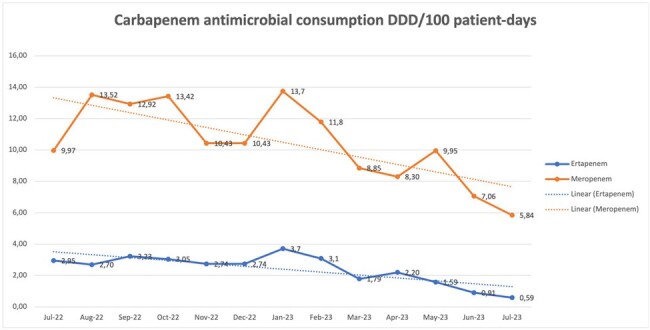

**Methods:**

A before and after study was conducted from July 2022 to July 2023, with a baseline period (July to December 2022) and an intervention period (January to July 2023). During the intervention, the ASP emphasized carbapenem prescription control while addressing other antibiotics as secondary concerns. Daily monitoring of meropenem and ertapenem prescriptions was prioritized, with other antibiotics monitored as resources allowed only when all carbapenem prescriptions were assessed. Selective reporting eliminated carbapenems from antibiograms in natural resistance patterns, and susceptibility testing for piperacillin-tazobactam was expanded to guide carbapenem-sparing therapies. Educational sessions focused on appropriate carbapenem use

**Figure:**
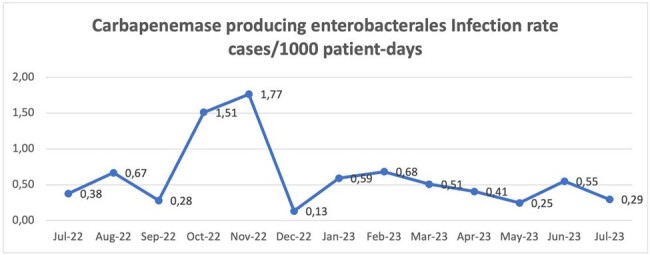

**Results:**

A decrease in the consumption of carbapenems was observed between the baseline period and the intervention period going from 13.52 (DDD/100 patient days) to 5.84 for meropenem and from 2.70 to 0.59 for ertapenem. Chart1. Additionally, a decrease in the infection rate was observed going from an average of 0.79 (cases/1000 patient days) in the baseline period to an average of 0.47 in the intervention period. Chart 2. The prevalence of carbapenemase producing enterobacterales did not change between the pre and post intervention period but the infection rate did. Chart 3. The non supervised use of other antibiotics was not related to an increase in their consumption. Chart 4.

**Figure:**
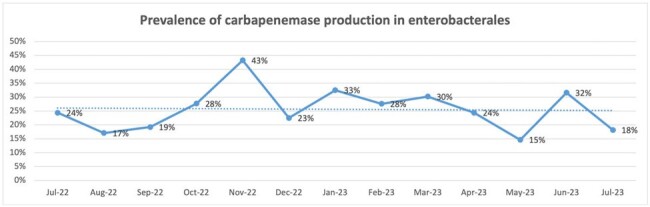

**Conclusion:**

ASP strategies can be directed toward a specific antimicrobial group in the pursuing of controlling specific problems such as carbapenem overuse and carbapenemase-producing-enterobacterales. These directed strategies can be implemented without an increase in the consumption of other antimicrobial groups.

**Figure:**
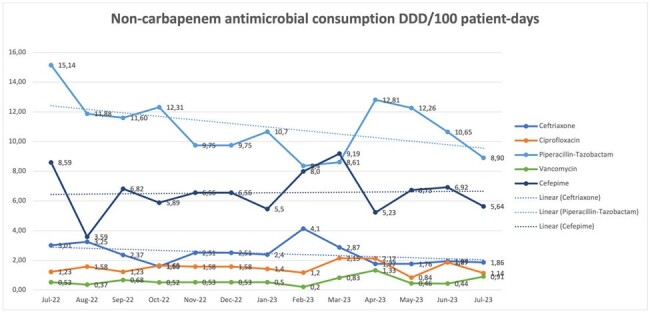

**Disclosures:**

**All Authors**: No reported disclosures

